# Development and Characterization of a Novel Low-Cost Water-Level and Water Quality Monitoring Sensor by Using Enhanced Screen Printing Technology with PEDOT:PSS

**DOI:** 10.3390/mi11050474

**Published:** 2020-04-30

**Authors:** Bei Wang, Manuel Baeuscher, Xiaodong Hu, Markus Woehrmann, Katharina Becker, Nils Juergensen, Moritz Hubl, Piotr Mackowiak, Martin Schneider-Ramelow, Klaus-Dieter Lang, Ha-Duong Ngo

**Affiliations:** 1 University of Applied Sciences Berlin, Wilhelminenhofstr. 75A, 12459 Berlin, Germany; Xiaodong.Hu@HTW-Berlin.de (X.H.); Katharina.Becker@HTW-Berlin.de (K.B.); Moritz.Hubl@HTW-Berlin.de (M.H.); 2 Fraunhofer Institute for Reliability and Microintegration IZM, Gustav-Meyer-Allee 25, 13355 Berlin, Germany; Manuel.Baeuscher@izm.fraunhofer.de (M.B.); Markus.Woehrmann@izm.fraunhofer.de (M.W.); Nils.Juergensen@izm.fraunhofer.de (N.J.); Piotr.Mackowiak@izm.fraunhofer.de (P.M.); Martin.Schneider-Ramelow@izm.fraunhofer.de (M.S.-R.); Klaus-Dieter.Lang@izm.fraunhofer.de (K.-D.L); 3 Center of Microperipheric Technologies, Technical University Berlin, Gustav-Meyer-Allee 25, 13355 Berlin, Germany

**Keywords:** water-level sensor, water quality monitoring, adhesive peeling force test, screen printing, poly(3,4-ethylenedioxythiophene):poly(styrenesulfonate) (PEDOT:PSS), conductive polymer, capacitive sensor

## Abstract

A novel capacitive sensor for measuring the water-level and monitoring the water quality has been developed in this work by using an enhanced screen printing technology. A commonly used environment-friendly conductive polymer poly(3,4-ethylenedioxythiophene):poly (styrenesulfonate) (PEDOT:PSS) for conductive sensors has a limited conductivity due to its high sheet resistance. A physical treatment performed during the printing process has reduced the sheet resistance of printed PEDOT:PSS on polyethylenterephthalat (PET) substrate from 264.39 Ω/sq to 23.44 Ω/sq. The adhesion bonding force between printed PEDOT:PSS and the substrate PET is increased by using chemical treatment and tested using a newly designed adhesive peeling force test. Using the economical conductive ink PEDOT:PSS with this new physical treatment, our capacitive sensors are cost-efficient and have a sensitivity of up to 1.25 pF/mm.

## 1. Introduction

Sensors and microsystems appear widely in different areas, especially in this explosively growing digitalization era. With further developments and studies on material science, this field has many more divisions and refinements due to all kinds of purposes and working environments. Unlike conventional semiconductor sensors, the new generation of sensors and microsystems focus more on environment-friendly, flexible, and cost-efficient materials and processes, such as conductive polymers and flexible devices [1,2,3].

Water-level and water quality sensing have a wide application in both industrial and environmental monitoring. When the pollutants change the water quality, sensors can measure this change by detecting the surrounding electromagnetic field. Paczesny et al. [4] reported a silver nanoparticle printed capacitive sensor by using inkjet printing technology, with a sensitivity of 0.074 pF/mm. Due to the satellite droplet, the inkjet printing technology enlarges the chance of conductive failure of electrodes. Based on [4], using inkjet printing technology, Yang et al. [5] improved the sensitivity of printed sensors to around 1.9 pF/mm in test fluids of high ion concentrations. However, the conductive material using silver nanoparticles has been proved highly toxic [6,7,8]. Even a small leak of silver nanoparticles from the printed sensor could pollute water sources seriously. More environment-friendly materials can be used.

One of the biocompatible conductive polymers is the poly(3,4-ethylenedioxythiophene) (PEDOT) [9]. Thanks to its electrical conductivity and environmental stability [10,11,12,13], PEDOT is widely used in many kinds of applications, such as the discharge in the fabrication of OLEDs [14,15], electrochromic devices [16,17] and sensors [18,19,20,21,22], etc. The most common use of PEDOT in the market is a complex of PEDOT and poly(styrenesulfonate) (PSS), named PEDOT:PSS. It has the advantage of being applied as conductive ink in printed electronics. The hydrophobic PEDOT is dispersed into the hydrophilic PSS. In PSS, the sulfo group acts as a soluble matrix for the PEDOT domain and helps to maintain the electrical neutrality by introducing counter ions. This morphological feature results in adhesive bonding with sub-layers and also a lower electrical conductivity of about 1–10 S/cm [23].

There are few pieces of literature on the measurement of the adhesive bonding force for printed electronics. In 2019, Woehrmann et al. proposed a method of the adhesive peeling test for measuring the bonding force of thin film [24], but it cannot be applied for the bonding test of printed electronics directly. In 2019, Xu et al. mentioned an adhesive peeling test measurement [25] but did not provide many details about the measurement setup and the dependence on the peeling angle and peeling speed.

Moreover, the sheet resistance of the PEDOT:PSS is 190 Ω/sq given by the datasheet [26]. The conductivity of the PEDOT:PSS is particularly affected by different printing and curing methods, which can limit its potential as an electrically conductive material. Several methods have been proposed to improve the conductivity of the PEDOT:PSS, such as treatment with various nanocomposites [14,27,28], which is rather expensive both in material and processing, and chemical treatment using solvents [23,29,30].

In our work, we developed our sensor using this biocompatible PEDOT:PSS. A simpler physical treatment by using a modified multilayer printing process was performed. Together with the screen printing technology, the conductivity of this PEDOT:PSS ink was largely improved. The chemical bonding of the interface between the printed ink and the substrate were studied. A simple chemical treatment to the substrate was performed to increase the bonding force in between the substrate and the PEDOT:PSS ink. Section 2 will provide details of the methodology, including the sensor design, processing, characterization, adhesion peeling test, and capacitive measurement setup. The results and discussion of the thickness of printed layers, sheet resistance, printed sensor, adhesion peeling test, and the capacitive sensing on different water-levels and pH values will be presented in Section 3. This work concludes in Section 4.

## 2. Methodology: Processing and Characterization

The contents of this methodology section are briefly mentioned here to give an overview. Section 2.1 is about sensor design. The principle that a good conductivity of printed electrodes facilitates the better performance of capacitive sensing is illustrated here using a simplified lumped-element model. Section 2.2 presents the processing workflow of the enhancement to improve the conductivity of the printed electrodes. After that, in Section 2.3, the bonding force between printed PEDOT:PSS and polyethylenterephthalat (PET) substrate is investigated and improved via simple chemical treatment. In Section 2.4, the characterization includes the thickness of printed layers, the sheet resistance, cross section morphology, and the adhesion bonding force for which a modified adhesion peeling force test was established and analyzed. For more stable capacitive sensing, a capacitive measurement setup is presented in Section 2.5.

### 2.1. Sensor Design

**Figure 1:**
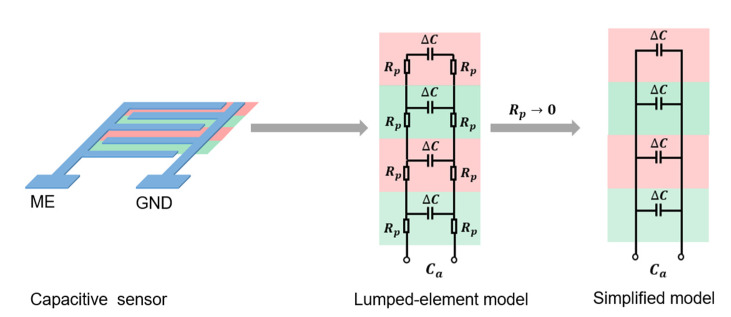
Lumped-element model of capacitive sensing and its relation with the resistance.

Capacitive sensors belong to the proximity sensing technology. They have been widely used in industries and daily life [31,32,33,34] due to their advantages in facile fabrication, effective cost, and quick response. A capacitive sensor works basically like an open capacitator, which forms an electromagnetic field between and surrounding the measuring electrode (ME) and the ground electrode (GND), such as the sensor shown in Figure 1. It detects the nearby objects by sensing whether the field distribution has been changed.

A commonly used model for dimensioning purposes of interdigital capacitive sensor systems was developed and reported by Abu-Abed et al. [35]. Our previous work further modeled and simulated the interdigital structure and the capacitance, as reported by Baeuscher et al. [18]. Based on these, we newly designed the printed PEDOT:PSS sensors for water-level and water quality monitoring sensing in two different structures, i.e., 1 and 1.5 mm electrode width. The spacing between the interdigital electrodes was set identical to the electrode width of each sensor. All the printed electrodes were designed to be connected with 3-mm width printed conductors on both sides. The novel organic, biocompatible, conductive screen printing ink PEDOT:PSS PE-EL-P5015 (from AGFA ORGACON, Morzer, Belgium, viscosity >50 Pa·s) [26] was used as our electrode material. The electric conductivity of the PEDOT:PSS was indicated by measuring sheet resistance *R*_sq_. The sheet resistance of a screen printed single layer was measured as having a mean value of 264.39 Ω/sq. However, the conductivity is not good enough for effective sensing.

Better conductivity leads to more effective capacitive sensing. As shown in Figure 1, a brief expression was made by applying the lumped-element model. In the lumped-element model, the capacitive sensor was modeled by several identical units highlighted in pink and green in Figure 1. If we ignore the effect of parasitic inductance and parasitic capacitance, each unit can be modeled as an electrical circuit with the measuring capacitance ∆C and parasitic resistances $R_{p}$, along both printed electrodes. The units were connected in parallel to their adjacent units. The entire sensor was measured at the end of the modeled circuits. The measured capacitance of the sensor is defined as $C_{a}$.

According to the Kirchhoff laws, under ideal conditions, $C_{a}$ is expected to be the sum of capacitance ∆C of each unit, as shown by the simplified model in Figure 1. In actual fact, the parasitic resistances do affect the measured capacitance, as shown by the lumped-element model in Figure 1. Only if the parasitic resistance becomes negligibly small would the measured capacitance become close to the expected value in the simplified model, which has no measurement error caused by the parasitic resistance $R_{p}$. Therefore, to bring the measured capacitance $C_{a}$ closer to the ideal expected value and lower the measurement error, the parasitic resistance $R_{p}$ needs to be reduced. This means that the conductivity between printed electrodes needs to be improved, which could be achieved via the enhancement in Section 2.2.

### 2.2. Processing

An enhanced multilayer printing process has been performed to improve the conductivity of printed PEDOT:PSS. Challenging problems always appear during the multilayer printing process of mm- or even μm-scale structures, especially the processing starting from the second layer. For instance, both the spread-out of inks before curing and the calibration of layers after curing lead to damage of printed designed structures. These problems are due to the high viscosity of inks and high surface tension. In this work, the PEDOT:PSS printed structures were partially-cured during the printing process in order to approach a semi-crystalline state. This state has lower surface energy compared with before curing, which can facilitate further printing of multilayers.

**Figure 2:**
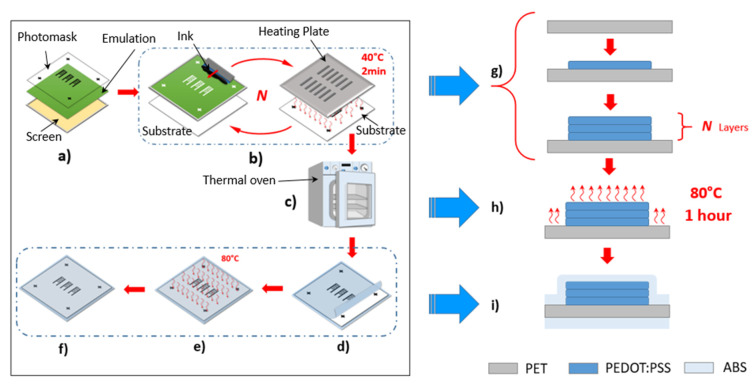
The workflow for multilayer screen printing: (**a**) preprocess: preparing the screen; (**b**) multilayer printing process; (**c**) curing process; (**d**–**f**) the coating process. The illustration of printing in cross section: (**g**) the printing process corresponding to steps (**a**,**b**); (**h**) the curing process corresponding to (**c**) and (**i**) the protective coating process corresponding to (**d**–**f**).

Our process workflow, as presented in Figure 2, contains four main important steps. In the first step, the preprocess, a photomask with designed sensors covered the UV-light-sensitive emulation layer on the stencil of a screen. The part of the screen beneath the designed structure was blocked off during the exposure to UV-light. Only the blocked area was not cured by the UV-light and was dissolvable in water, which opened the corresponding spaces on the emulation layer. The ink (PEDOT:PSS) could penetrate through these spaces on the substrate surface during printing. In the second step, the printing process, a heating plate was used to heat the printed ink for 2 min at 40 °C to shape the sensors preliminarily. The increasing molecular thermal activity reduced the cohesive force inside the liquid ink, thereby lowering the surface tension. This heating process ensured sharper boundaries of printed structures and also prevented the sticking of the printed ink during the iterations of printing. In the third step, the curing process, the printed sensors were cured in a thermal oven for 1 h at 80 °C, as described in previous work [18]. In the fourth step, the coating process, for protection purposes, the cured sensors were coated with acrylonitrile butadiene styrene (ABS) layers by applying a laminator at 80 °C. The protective ABS layers can isolate the oxygen, prevent chemical reactions, slow down the aging of printed ink, and protect the sensors from mechanical damage, such as scratches and frictions.

### 2.3. Bonding and Chemical Treatment

Like the conventional sensor manufacturing process, the performance of the printed sensor also depends on reliable bonding between the functional layers and the substrate.

**Figure 3:**
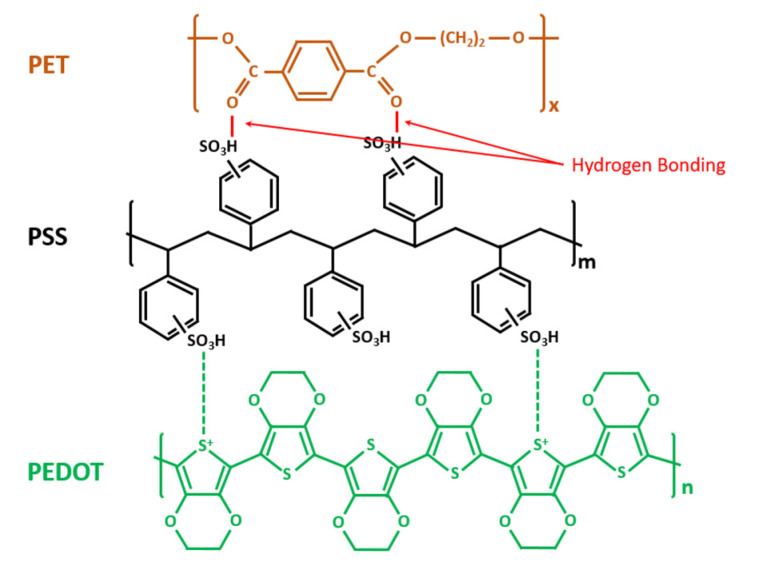
Chemical structures of poly(3,4-ethylenedioxythiophene):poly(styrenesulfonate) (PEDOT:PSS), polyethylenterephthalat (PET) and their chemical bonds.

**Figure 4:**
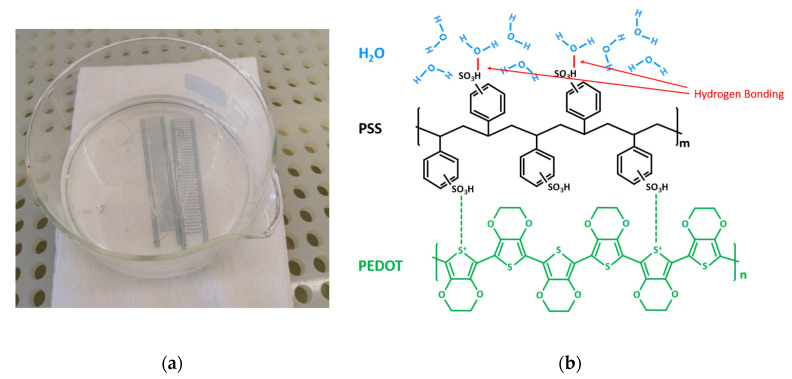
(**a**) Hydrogen bonding test; (**b**) the schematic of hydrogen bonding between PEDOT:PSS and deionized (DI) water.

Studying the chemical structures of PEDOT:PSS ink and the PET substrate as shown in Figure 3, we suppose that the bonding between PEDOT:PSS and PET is mainly hydrogen bonding. As the hydrophobic PEDOT dispersed into the hydrophilic sulfo group PSS, the hydrogen of the PSS group bonded with the oxygen in PET, forming the hydrogen bonding at the interface of the materials. Figure 4a shows a hydrogen bonding test in the cleanroom. During this test, two cured PEDOT:PSS printed sensors were placed into deionized water (DI water) for 5 h. After that, the PEDOT:PSS ink could be easily separated from the PET substrate. This indicated that the hydrogen in the PSS group bonded priorly with a large amount of oxygen in water molecules, thus lessening the hydrogen bonding between the PEDOT:PSS and PET substrate, as shown in Figure 4b. The bonding force, therefore, became much weaker, and the reliability of the printed sensors was reduced. A much larger bonding force is needed for more reliable printed sensors.

To increase the bonding force between the PEDOT:PSS and PET substrate, a chemical treatment to the PET substrate was performed with a solvent which has 10 wt % chloroform (CHCl_3_) mixed with 90 wt % ethanol (concentration: 95%). In this solvent, the ethanol functioned as a stabilizer and the chloroform as the activator. The PET substrate was first painted with this solvent and laid still for 20 min in a ventilated environment at a temperature of 21 °C. After that, the chemical-treated PET was washed with ethanol and dried.

### 2.4. Characterization

**Figure 5:**
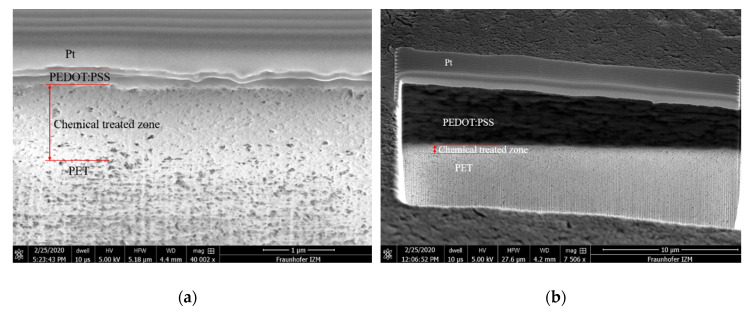
SEM cross section images of (**a**) single-layer printed PEDOT:PSS and (**b**) of a 10-layer printed PEDOT:PSS.

After the chemical treatment mentioned in Section 2.3, a chemical-treated zone was created between the printed layer and the substrate to increase the bonding force. Figure 5 shows the scanning electron microscope (SEM) images of the cross section view of a printed single layer and 10-layer sample. Under the platinum (Pt) deposited sacrificial layer, the cured PEDOT:PSS was stacked layer by layer on the PET substrate. The interfaces between the chemical-treated zone and the PET substrate were not clearly observable. After investigation by using the focus ion beam (FIB) and SEM, the thickness of the chemical-treated zone was measured to be about 800 nm on average, the thickness of the printed single layer was measured to be 325.2 nm and that of the printed 10-layer was measured to be 5.623 µm on average. The sheet resistance is also an essential characteristic of the printed sensors. The measured sheet resistance of different numbers of layers will be shown in the results in Section 3. Another important characteristic is the bonding force. To measure the bonding force between the printed PEDOT:PSS and its PET substrate, a modified adhesive peeling test was designed and performed, which is shown next.

#### Adhesive Peeling Force Test

**Figure 6:**
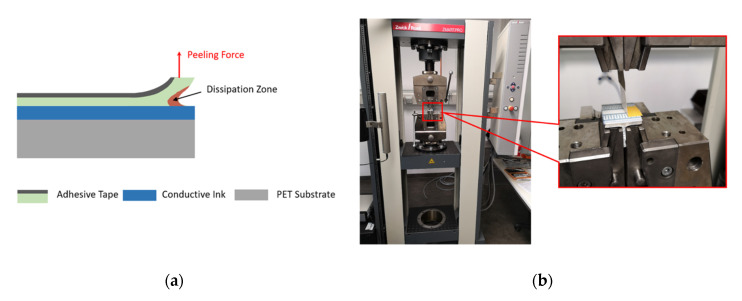
Schematic. (**a**) The peeling force test; (**b**) the entire device-under-test (DUT) and test machine in the laboratory.

The bonding force between printed structures and the substrate needs to be tested to ensure the reliability and functionality of sensors. Generally, the peeling force measurement is perpendicular to the device-under-test (DUT) at a fixed angle of 90°, as shown in Figure 6a. Practically in our test setup using the tensile test machine Zwick/Roell^®^ (Zwick Roell Group, Ulm, Germany) as shown in Figure 6b, the angle between the adhesive tape and the DUT was unfixed and rose as the movable arm went up. Therefore, the expected peeling force was the perpendicular part of the measured force and could be separated from the measured result mathematically.

**Figure 7:**
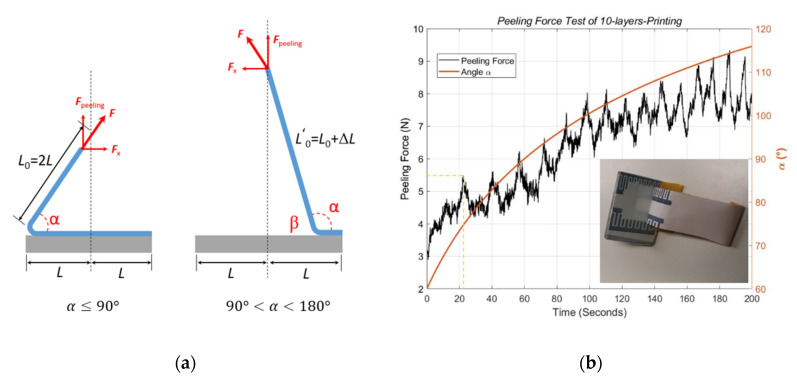
Schematic: (**a**) peeling force test related to angle α. The tape is of length 4 L. (**b**) Measured results of a 10-layer printed sample at a constant speed of 10 mm/min.

The test was performed with a strong double-sided adhesive tape Tesa^®^ 56171 (Tesa, Hamburg, Germany). In the preparation phase, the adhesive tape was glued on the surface of the printed sample and put under a uniform pressure for 5 min in order to obtain sufficient adhesive contact. We took a piece of the tape of length 60 mm. Half of the tape was fixed for the adhesive contact segment, and the remaining half was set free for the test machine. This entire DUT was fixed on a t-shaped aluminum holder, which was later clamped tightly by the lower side in the test machine, as illustrated in Figure 6b. The upper side in the test machine was the measuring arm, which could move in the vertical direction. The free end of the tape was then fixed onto the tension clamp, which was bonded with the measuring arm. During the test, with the measuring arm moving up perpendicularly at a constant speed, the adhesive tape was separated from the surface of the printed samples gradually at varying angles to the test DUT, as shown in Figure 7a.

As illustrated in Figure 7a, the peeling force $F_{peeling}$ is only the perpendicular part of the measured force F. Moreover, $F_{peeling}$ is highly related to the angle α. Here, we define it mathematically as

(1) $$F_{peeling}=F\cdot sin\alpha$$

The process of the adhesive peeling test is analyzed in two phases, α≤90° and 90°<α<180°.

Let the moving speed of the movable arm be v. The free end of the adhesive tape has a length of $L_{0}=2L$. In the first phase α≤90°, we describe the relation of the movement (v·t) of the adhesive tape and the angle α as $cos\alpha =\frac{L-v\cdot t}{L_{0}+v\cdot t}=\frac{L+L_{0}}{L_{0}+v\cdot t}-1$. Here, t is the measurement time (seconds). From the formula for cosα, the angle α can be calculated as

(2) $$\alpha =\arccos\left(\frac{3L}{2L+v\cdot t}-1\right)$$

Substituting Equation (2) into (1), we get the peeling force as

(3) $$F_{peeling_{,}\alpha \leq 90{}^{\circ}}=F\cdot sin\alpha$$

In the second phase, 90°<α<180°, let β=180°−α. We describe the relation of the movement (v·t) of the adhesive tape and the angle β as $cos\beta =\frac{v\cdot t-L}{L_{0}+v\cdot t}=1-\frac{L+L_{0}}{L_{0}+v\cdot t}$. From the formula for cosβ, the angle β is calculated as

(4) $$\beta =\arccos\left(1-\frac{3L}{2L+v\cdot t}\right)$$

Substituting Equation (4) into (1), we obtain the peeling force as

(5) $$F_{peeling_{,}90{}^{\circ}<\alpha <180{}^{\circ}}=F\cdot sin\beta$$

The measured force F needs to first be calibrated with Equations (3) and (5) to obtain the peeling force, which is perpendicular to the DUT during the test. Figure 7b shows the calibrated results of the peeling force and the corresponding angle α during the test procedure. As the testing arm moving up vertically at a constant speed, the angle α increased smoothly during the measurement. Our mathematical model above describes this process exactly, including the smooth change in angle α with respect to the rising movable arm. The peeling force $F_{peeling}$, which is highly correlated with angle α, can be separated from the measured force F.

**Figure 8:**
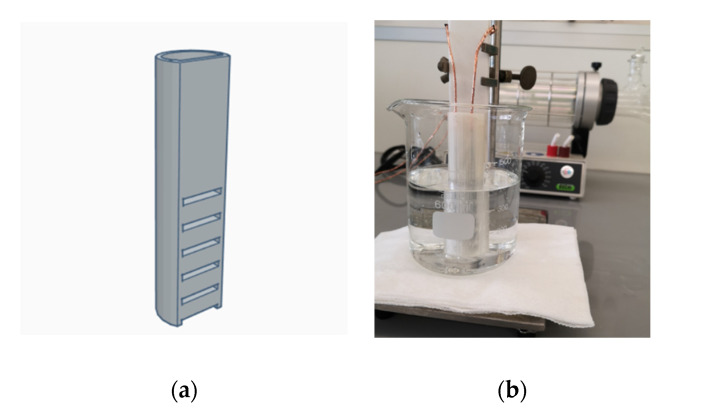
(**a**) Three-dimensional design of the sub-environment container; (**b**) measurement setup of capacitive water sensing.

The performed force of the test machine was raised in an undulating way to damage the bonding between the printed ink and the substrate. In the first 20 s, the peeling force increased without significant peaks, and none of the printed ink was peeled off from the DUT. After that, the peeling force swung during the pulling up process until the printed ink was finally removed from the substrate. The peaks of the curve in Figure 8b represent the peeling force at the interface of the adhesive tape and printed PEDOT:PSS, and the valleys indicate the peeling force at the interface of the adhesive tape and the PET substrate. Therefore, the number of peaks corresponds to the number of electrodes. As shown in Figure 7b marked by the yellow dashed lines, the stripping process began at the 22nd second at the angle α = 87°, and the peeling force was about 5.5 N. After stripping for about 180 s, the adhesive tape was peeled off for a length of 30 mm. Together with 25 mm width of the adhesive tape, the entire test area was 30 mm × 25 mm.

### 2.5. Capacitance Measurements Setup

The measurement was performed by using the capacitance-to-voltage-converter IC PCAP04 (AMS AG, Premstaetten, Austria) with 7.5 V power supplied connected to a PC. The cycle clock frequency of the converter IC PCAP04 was set to 50 kHz (default working frequency), and the measuring rate was 12.5 Hz. An external 10 pF capacitance was applied as the reference capacitance.

Essentially, the measured capacitance is affected by the electromagnetic field surrounding the electrodes. The printed capacitive sensor and converter IC were connected by two wires, which must be shielded by grounded metal cladding in order to eliminate the electromagnetic interference in between. In addition, capacitive sensing is highly susceptive to its working environment. Even fluid vibrations can cause significant measurement errors. To eliminate the effect of fluid vibrations, we designed a long and narrow shaped container of the size of our sample sensors with open windows on the side, as shown in Figure 8a. Next, we placed the sample sensor into the container, and then put the container into a 500 mL volume glass beaker. We created a sub-environment connected with the test fluids by using the container, which could stabilize the fluid vibrations around the sensor. The material of the container is chemically inert to our test fluids. Although both the container and the glass beaker can cause parasitic capacitance, the former can be eliminated via calibration, and the latter is negligibly small. Figure 8b shows the entire measurement setup.

## 3. Results and Discussion

### 3.1. Thickness, Sheet Resistance, and Sensor Sample

**Figure 9:**
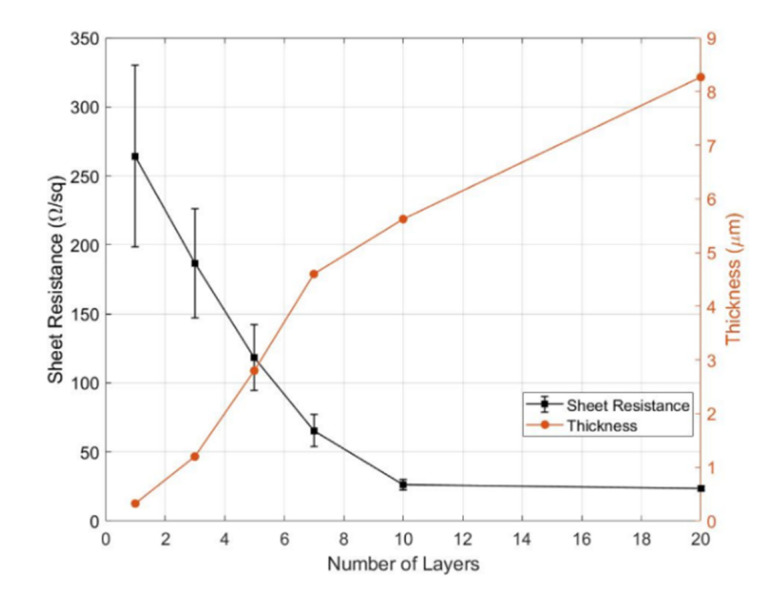
The sheet resistance and thickness with respect to the number of layers.

After the physical enhancement in Section 2.2, the thickness of the printed area increased, and as a result, the sheet resistance declined dramatically. Our experimental results are shown in Figure 9. The thickness of the printed area for a single layer was 325.2 nm and increased to 8.26 µm for 20 layers, while the sheet resistance for a single-layer printed area was about 264.39 Ω/sq and sank to about 23.44 Ω/sq for a 20-layer printed area. Meanwhile, the error deviations declined from ±25% at a single layer to negligibly small at 20 layers. This decreased error indicates that the charge transfer inside the printed areas became more stable. Particularly from 10 to 20 layers, the sheet resistance only slightly reduced, from 26.23 to 23.44 Ω/sq, by about 10%, whereas the thickness increased further from 5.623 to 8.267 µm by about 47%. These results reveal that the cost of printing more than 10 layers gained little benefit in decreasing the sheet resistance. Moreover, the charge transfer remained stable after the thickness reached 5.623 µm, which corresponded to the thickness of 10 layers in our case.

**Figure 10:**
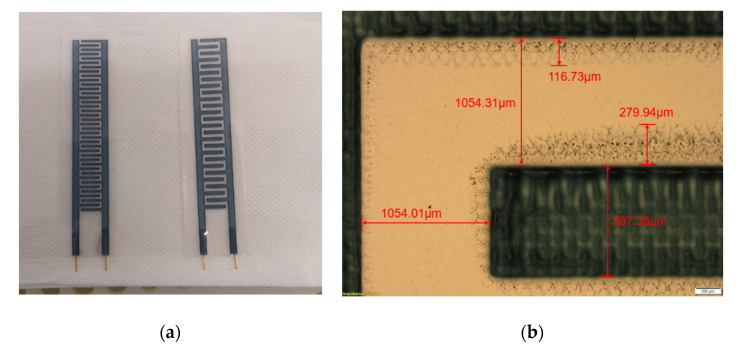
(**a**) Printed samples of PEDOT:PSS capacitive sensors; (**b**) photo image of 1-mm electrode sensor.

Based on the results above, the designed capacitive sensor was printed in 10 layers and later coated with 80 µm-thick ABS after the curing process. The ABS is stable in water, acid, and alkali. Thus, the coated sensor was well protected during the test, and after the test could be cleaned safely with the ethanol solution (see Figure 10a).

Figure 10b shows the details about the 1-mm-width electrode sensor. The printed interdigital electrode was measured to be 937.35 µm in width uniformly, which has −6.2% error from the designed 1 mm. The spacing in the vertical and horizontal directions was 1054.31 and 1054.01 µm, respectively, which were slightly deviated from the defined 1 mm spacing.

Around the printed area, there were also the overflow areas that occurred during the printing process simultaneously. These overflow areas were mainly caused by the pressure on the ink during the printing. The overlapping of the overflow areas between neighboring electrodes could cause electrical short circuits and measurement errors. In the worst case, it could damage the sensor. With 1-mm-width spacing between neighboring electrodes, the overflow area was slightly below 300 μm. This spacing avoided overlapping overflow areas between neighboring electrodes and prevented its instability during the sensing process.

### 3.2. Adhesive Peeling Force Results

**Figure 11:**
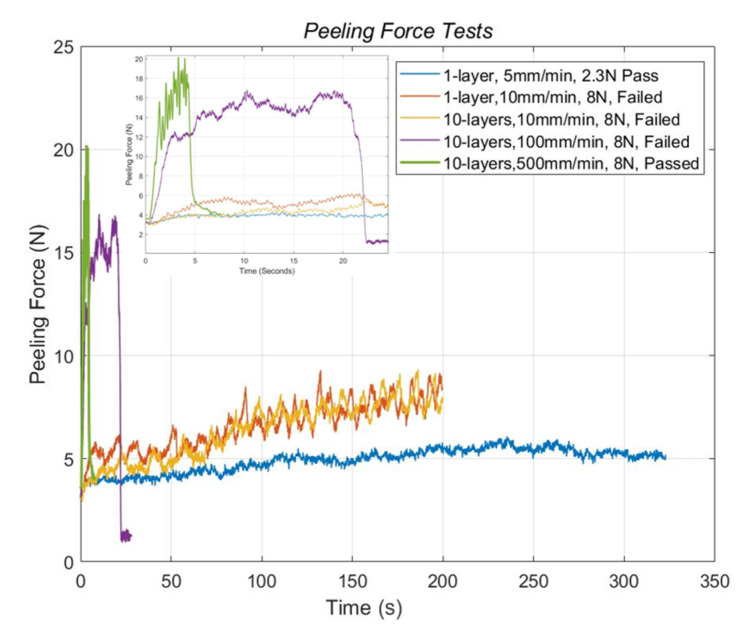
Peeling force tests of printed samples on non-chemical-treated (1-layer samples) and chemical-treated substrates (10-layer samples).

The bonding force and the bonding reliability of the PEDOT:PSS printed samples on the non-chemical-treated and chemical-treated PET substrate were tested. In the first case, as shown in Figure 11, a single-layer printed sample on the non-chemical-treated substrate was tested with the processing speed at 5 mm/min (marked with the blue curve). After raising the peeling force to 5.3 N, the one-layer sample was not separated from the substrate. After that, the sample was further tested at a speed of 10 mm/min. At this time, the bonding was damaged at a larger force 7.32 N, as shown by the orange curve in Figure 11. To compare the effects from different speeds, the PEDOT:PSS 10-layer samples printed on the chemical-treated PET substrate were tested at various speeds in the adhesive peeling tests. The yellow and violet curves represent the tests at the speed of 10 and 100 mm/min, respectively. In both cases, the bonding between the printed PEDOT:PSS and PET substrate was not damaged. Notably, the peeling force at the speed of 100 mm/min were increased to 16 N. In the extreme case at the maximum speed of 500 mm/min; the peeling force increased from 12 to 20 N as the green curve represented, the printed structure was still not peeled off from the substrate. These results indicate that our chemical treatment largely enhanced the bonding rate of hydrogen bonds. This enhanced boding rate is related to the increase in free hydroxyl and carboxyl groups on the chemical-treated substrate surface. These results show that the bonding became strong enough for making PEDOT:PSS printed sensors on PET substrate more reliable.

### 3.3. Capacitance Sensing

**Figure 12:**
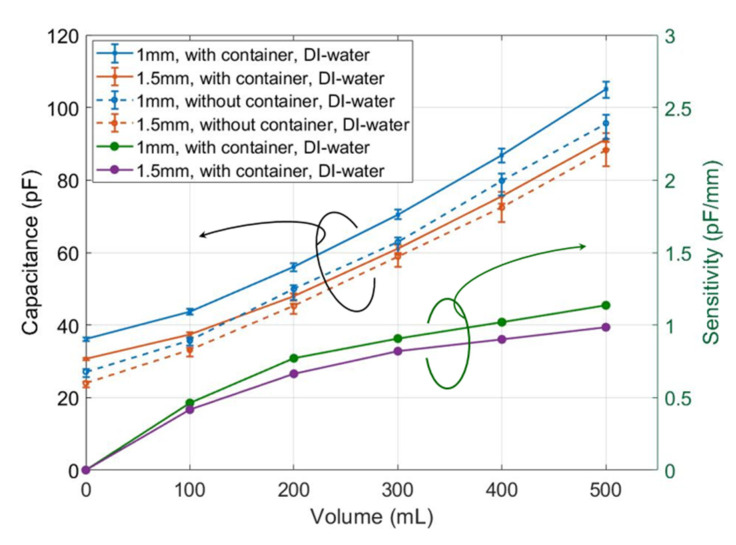
Capacitance measurement of sample sensors with and without container in DI water, and the related sensitivity.

We measured the sensors of 1 and 1.5 mm finger width, with and without the sub-environment container in DI water, respectively. The measured results, as shown in Figure 12, were used later as the reference for other test fluids.

Usually, the measured capacitance increases with rising water-level, which in our glass beaker is 1.6 cm per 100 mL. The capacitance of the 1-mm sensor using the container increased from 36.28 pF at 0 mL to 105.03 pF at 500 mL. Meanwhile, that of the 1.5 mm-sensor using the container increased from 30.68 pF at 0 mL to 91.27 pF at 500 mL, as shown by the blue and orange solid lines in Figure 12, respectively. The blue and orange dashed lines in Figure 12 show the measured capacitance of 1- and 1.5-mm sensors when there was no container and the volume increased from 0 to 500 mL. The solid and dashed lines (either blue or orange) have the same trends but with an average of 7.5 pF offset. This offset was caused by the container and was eliminated via a calibration without affecting the water-level testing. Moreover, the error ranges on the solid lines are much smaller than on the dashed lines, which verifies the stabilizing effect of using the container.

The sensitivities of the measured results of 1- and 1.5-mm sensors with containers were calculated and shown by the green and violet lines in the bottom left of Figure 12. The sensitivity of the sensor was calculated as the measured capacitance change per volume change. For the 1-mm sensor, as the solid blue line shows, the measured capacitance changed from 36.6 to 44 pF as the volume increased from 0 to 100 mL. Then the sensitivity of the 1-mm sensor has a sensitivity of 7.4 pF/100 mL at the volume of 100 mL. Based on the marker 1.6 cm per 100 mL on the beaker, it is 0.46 pF/mm, as the green line shows. At 500 mL, the sensitivity rose to 18.8 pF/100 mL (1.13 pF/mm). Compared with the violet line, the sensitivity of the 1-mm sensor is about 10–15% higher, which means the 1-mm sensor has a higher sensitivity than the 1.5-mm sensor. Currently, we found no other published results using PEDOT:PSS. Water-level sensors by [4] using a different printing method and the material Ag nanoparticle has a sensitivity of 0.074 pF/mm, which is much lower than ours. While based on [4], Yang et al. [5] largely improved the sensitivity to 1.9 pF/mm, which is higher than ours. Due to the small sensor size with fine electrodes and the inkjet-printing technology of [5], their processing and material were quite expensive.

These results showed that the measurement error of a sensor using the container was smaller than not using the container, and the sensitivity of the 1-mm sensor was higher than that of the 1.5-mm sensor. The extra offset in the measurement brought in by the container was calibrated afterward. We chose the 1-mm sensor with the container for the following water quality measurements. Three chemical buffer solutions with distinct pH values were used as test fluids, i.e., the citric acid/sodium hydroxide/hydrogen chloride (pH at 20 °C = 2.04), phosphate (pH at 20 °C = 7), and borate-HCl (pH at 20 °C = 8.74).

**Figure 13:**
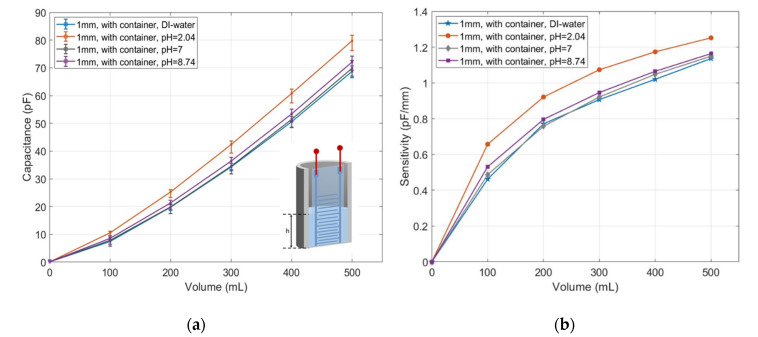
(**a**) Measured capacitance in response to the deionized water and buffer solutions with pH values of 2.04, 7 and 8.74; (**b**) their sensitivities.

The measured capacitance was first calibrated to eliminate the offset caused by the container, as shown in Figure 13a. The calibrated capacitance for pH = 2.04 increased from 0 pF at 0 mL to 80 pF at 500 mL, which was about 12 pF more than the reference fluid. The capacitance response to the solution of pH = 8.74 was 0 pF at 0 mL and increased to 74 pF at 500 mL, which was about 2 pF higher than the reference fluid. The capacitance of pH = 7 was only slightly higher than the reference fluid. The sensitivity of the measured sensor in both acidic and alkaline solution was stronger than in the deionized water as shown in Figure 13b. The sensitivity of the sensor in the acidic solution approached 1.25 pF/mm at 500 mL. Meanwhile, the value for the deionized water was about 1.14 pF/mm. The sensitivities of capacitive sensing in the solutions pH = 7 and pH = 8.74 were both slightly below 1.16 pF/mm, which was a little higher than in the deionized water.

As shown in Figure 13, the measured capacitance varies with different pH values. More precisely, the pH value is theoretically defined as 10 based-logarithm of the molar concentration of the hydrogen ion concentration in solution. It means that the ion concentration of other chemical elements can also lead to capacitance changes, as reported in paper [26]. Principally, the capacitive sensor measurement is dependent on the dielectric constant of the solution. For instance, S. Gevorgian [36] provided a mathematical model showing the relation between the capacitance and dielectric constant by using coplanar capacitance. A decade later, A. Levy [37] developed a mathematical approximation of dielectric response of solutions of different ion concentrations. Yang et al. [5] also reported mathematically and experimentally that the capacitance measured in solution depends on the ion concentration inside the solutions. When the ion concentration is less than 10^−5^ M or greater than 10^−2^ M, the capacitance changes slightly. In this case, the dielectric constant no longer dominates the change in capacitance. It indicates that the accuracy of the capacitive sensing in solution also depends on a good understanding of the field theory in liquid, especially with different ions inside, which could be part of our future work. On the other hand, as a cost-efficient material, the conductive polymer PEDOT:PSS still needs to be further investigated and studied. Especially interesting are the sensing-related properties like sheet resistance, charge transport, and bandgap energy. More in-depth research and discussions are still required for its applications in printed electronics and thin film technology on the bonding mechanism and the material modifications, including surface treatment. With a more accurate and mathematically proved model, people can better control this material and apply it more precisely in different areas.

## 4. Conclusions

In this paper, we presented a multilayer screen printing technology to enhance the conductivity of the PEDOT:PSS printed on PET substrates. To obtain reliable capacitive sensing, we investigated that the chemical bond at the interface between printing ink and PET substrate is mainly the hydrogen bond. Through a simple chemical treatment, we have improved the adhesion to the PET substrate. The adhesion bonding force between the printed ink and the substrate was tested using a newly designed adhesion peeling test setup. Via further characterization of the thickness and sheet resistance of the printed multilayers, we found that using 10 layers was most effective for printed PEDOT:PSS. The adhesion bonding force of the 10-layer printed sample was increased to 16–20 N at 100–500 mm/min. Based on these data, the capacitive sensor was developed and applied in water level and water quality sensing. Here, the water quality was tested mainly with three distinct pH values, which are related to the ion concentration inside the solutions.

The sensitivity of our capacitive sensor varies from 1.14–1.25 pF/mm. Available work from Paczesny et al. [4] had a sensitivity of 0.074 pF/mm, based on which Yang et al. [5] reached a sensitivity of 1.9 pF/mm. Both [4,5] adopted ink-jet printing technology, ink containing metal, and relatively complicated treatments. The sensitivity of our sensor is already quite good compared with available work in [4,5] with respect to the material and processing cost. Using the non-metallic, biocompatible conductive ink PEDOT:PSS and the cost-effective enhancement, our sensors have great potential for further industrial applications.
